# Total Knee Arthroplasty in a Patient with Bilateral Congenital Dislocation of the Patella Treated with a Different Method in Each Knee

**DOI:** 10.1155/2015/890315

**Published:** 2015-01-31

**Authors:** Hajime Yamanaka, Taisei Kawamoto, Hiroshi Tamai, Munetaka Suzuki, Tatsuya Kobayashi, Yawara Eguchi, Hideyuki Nakajima

**Affiliations:** ^1^Department of Orthopaedic Surgery, National Hospital Organization Shimoshizu Hospital, 934-5 Shikawatashi, Yotsukaido, Chiba 284-0003, Japan; ^2^Department of Orthopaedic Surgery, Matsudo City Hospital, Matsudo, Japan; ^3^Miyako Orthopaedic Clinic, Chiba, Japan

## Abstract

We have operated total knee arthroplasty in a patient with bilateral congenital dislocation of the patella treated with a different method in each knee.

## 1. Introduction

Congenital dislocation of the patella (CDP) is a rare condition, the etiology of which is uncertain, and late presentation is usually accompanied with osteoarthritis [1, 2]. Lateral dislocation of the patella is the most typical physical finding, even in extension and flexion. The affected knee generally develops to valgus deformity. Total knee arthroplasty (TKA) is a valid and useful treatment for such patients [3]. Several cases of osteoarthritis with CDP successfully treated with TKA, with or without realignment of the extensor mechanism, have been reported [1, 4–8]. It is important to correct the valgus deformity and balance the soft tissue in TKA, but this makes surgery very difficult compared to common TKA.

Here, we present an unusual case of a patient with bilateral CDP with valgus gonarthrosis treated by TKA with distal realignment to reduce the dislocated patella of the left knee, while the right knee was treated without realignment of the extensor mechanism. The patient's walking ability was improved and he was satisfied with the level of pain relief.

The patient provided consent for data concerning this case to be submitted for publication.

## 2. Case Report

A 70-year-old man had a 5-year history of bilateral knee pain, which had gradually worsened over the last one year, along with reduced walking distance secondary to bilateral osteoarthritis of the knee. He had no difficulty in daily life activities during childhood or adulthood. He had worked as a barber and had no history of trauma. There was no relevant family history. He walked with two crutches since developing bilateral knee pain and could not negotiate stairs. Conservative therapy at another medical clinic, including injection of hyaluronic acid into both knees, was not effective.

When he first visited our institution, physical examination revealed conspicuous bilateral quadriceps atrophy. Both quadriceps were rated as having strength 3 on a muscle testing scale of 0–5. The patellas were palpable at the lateral side of the bilateral femoral condyle and had no mobility during flexion and extension. The proximal tibiae were rotated outward. Preoperatively, the passive range of motion in both knees was −10° to 130°. There was an extension lag of 45° at both knees. Effusions were present in both knees. The knees showed no signs of instability or ligamentous deficiency.

**Figure 1:**
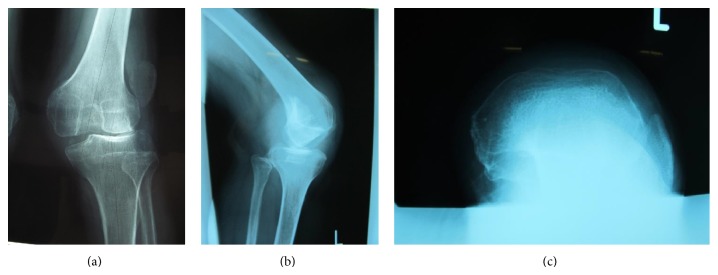
Preoperative radiographs: anteroposterior view (a), lateral view (b), and skyline view (c) of left knee.

**Figure 2:**
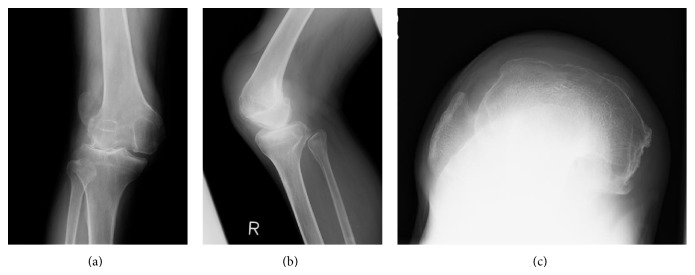
Preoperative radiographs: anteroposterior view (a), lateral view (b), and skyline view (c) of right knee.

**Figure 3:**
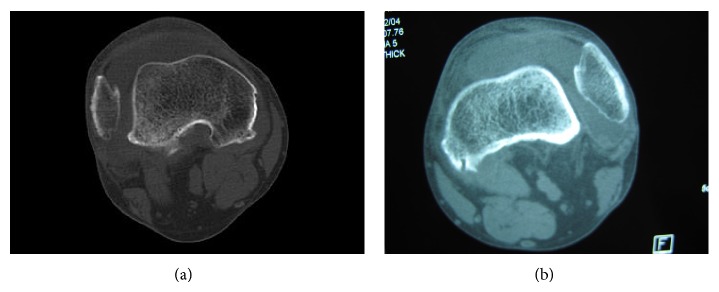
CT images of right (a) and left (b) knees.

Roentgenograms and computed tomography (CT) showed patellar dislocation and severe osteoarthritis of the bilateral knees with complete loss of the lateral compartment joint space (Figures 1–3). Valgus deformity of 10° was observed in each knee on standing with the lower extremities aligned. The patient had more pain in the left knee than the right knee. TKA with correction of the extensor mechanism was planned first for the more painful left knee.

**Figure 4:**
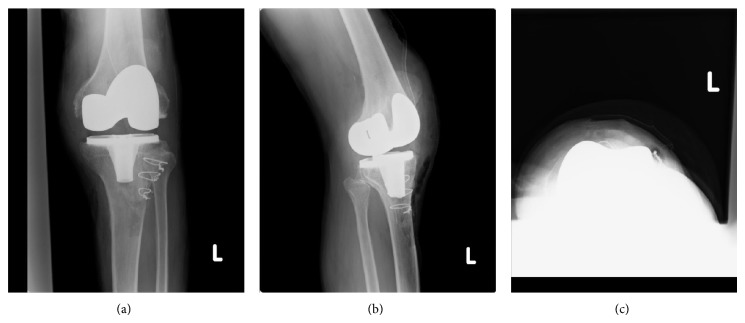
Postoperative radiographs: anteroposterior view (a), lateral view (b), and skyline view (c) of left knee.

A midline longitudinal skin incision was made under tourniquet control and lateral parapatellar arthrotomy was performed. The patella was located in the lateral gutter of the knee; the femoral condyle was hypoplastic. The medial retinaculum was thin. The vastus medialis oblique muscle was found to be located over the anterior aspect of the femur. For realignment of the extensor mechanism, we performed extensive medial and lateral retinacular release and distal realignment by tibial tubercle transfer. A posterior stabilized prosthesis (NexGen LPS-Flex; Zimmer, Inc., Warsaw, IN) was implanted and held in place with cement. Patellar resurfacing was not performed. The femoral component prosthesis was placed in 9° external rotation with reference to the posterior condyle line to facilitate patellar tracking. The iliotibial tract was partially detached from Gerdy's tubercle subperiosteally. The lateral collateral ligament and the popliteus tendon were also partially released from the lateral femoral condyle. The rotational alignment of the tibial component was based on the locations of the femoral components. After cementing the selected implants, the dislocated patella could not be reduced, so a proximal realignment procedure was performed as described by Insall et al. [9], and the rectus femoralis tendon was lengthened by Z-plasty. Thereafter, the patella was in the groove during extension, but passive flexion angle was limited to 90° of flexion of the knee with the hip flexed to 90°. The lateral side of the arthrotomy could not be closed. Medial plication was performed as possible as we could. Finally, the patella could not be reduced fully and did not track centrally, but more laterally, in the patellofemoral groove throughout the full range of movement (Figure 4).

Full weight-bearing walking was allowed 7 days postoperatively with a brace for 0° angle restriction for 2 weeks. Range of motion (ROM) exercise using a continuous passive motion device was started 1 week after the operation. At 4 weeks after the operation, ROM was 0°–90° in the left knee and the patient was discharged with one crutch.

**Figure 5:**
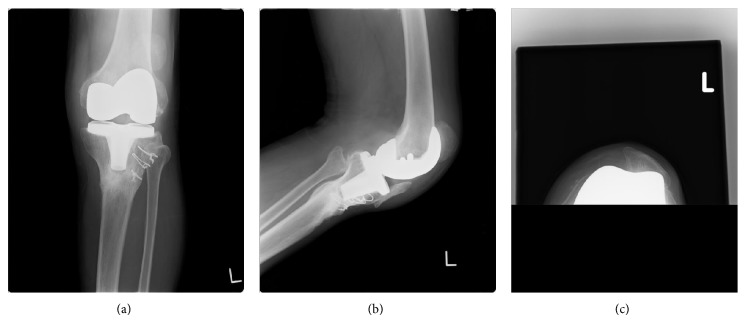
Final follow-up radiographs: anteroposterior view (a), lateral view (b), and skyline view (c) of left knee.

At 5 weeks after the operation, the patient slipped at home and fractured his left tibia. He was readmitted and conservative therapy was performed with a brace against the fracture. Three months later, his fracture was healed and he was discharged walking with one crutch (Figure 5).

One year after the operation on the left knee, he had hoped to undergo right knee surgery because of pain. He was not satisfied with the restriction of flexion angle of the left knee and was concerned about future fractures. Therefore, he asserted that it was not necessary to reduce the dislocated patella and realign the extensor mechanism in the right knee.

We performed TKA using the same prosthesis in the right knee without realignment of the extensor mechanism and reduction of the patella. A midline longitudinal skin incision was made under tourniquet control, and medial parapatellar arthrotomy was performed. The femoral component prosthesis was placed in 3° external rotation with reference to the posterior condyle line. The right patella was located on the lateral side of the femoral component.

**Figure 6:**
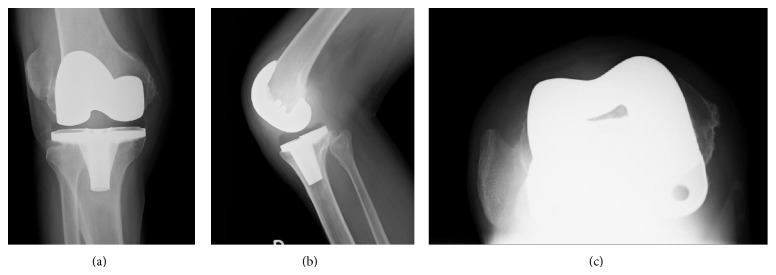
Final follow-up radiographs: anteroposterior view (a), lateral view (b), and skyline view (c) of right knee.

At the final follow-up one year after the right knee operation, the patient had full extension and 90° flexion in the left knee. There was an extension lag of 45° and flexion angle was 125° in the right knee. Left side quadriceps strength was improved to a rating of 4 on the muscle testing scale of 0–5, but that on the right side was not improved as before surgery. His walking ability was improved and he was satisfied with pain relief in both knees and walked with one crutch (Figure 6).

The preoperative Knee Society score and functional scores were 40 and 15, respectively, which improved to 83 and 60, respectively, at the final follow-up.

## 3. Discussion

CDP is a disorder of the knee joint on which the patella is permanently displaced, even in extension, and fixed on the lateral aspect of the femoral condyle. However, the etiology of CDP is still unknown [2]. In adulthood, gonarthrosis may develop mostly in valgus knees. There is no consensus regarding treatment of neglected elderly patients, but TKA is a useful therapy in CDP patients that have developed painful symptomatic osteoarthritis of the knee. A literature search revealed a limited number of similar cases in which CDP was treated with TKA. The first case treated by TKA was reported by Marmor in 1988 [7]. He did not relocate the extensor mechanism because it would have reduced the degree of flexion but had good results. Pradhan did not reconstruct the extensor mechanism in a case of CDP with TKA based on Marmor's report [6] but recommended that, in cases with excessive soft tissue release, a constrained type prosthesis should be considered because of instability of the knee. However, these reports did not present the component and functional results at long-term follow-up. Other authors corrected the extensor mechanism and relocated the patella, using lateral release and/or vastus medialis advancement with excellent results. No specific treatment protocols have yet been established for osteoarthritis in CDP.

Proximal or distal realignment is usually required to relocate the dislocated patella and the extensor mechanism during TKA. Proximal realignment of the extensor mechanism can be performed by Z-plasty or the Vulpius technique [3, 4, 6]. Dao et al. described a new technique of V-W quadricepsplasty in 2010 [2].

Tibial tubercle osteotomy is also effective for distal realignment, but with the risk of nonunion, soft tissue discomfort, and fracture [2, 3, 5]. In our case, left tibia fracture had occurred just below the site of tibial tubercle osteotomy. Therefore, we performed right side TKA without realignment of the extensor mechanism using osteotomy. If using osteotomy, it will be necessary to have a long extension stem of the tibial tray to prevent such fractures. Reddy and Kondreddi reported a two-stage procedure consisting of patellar realignment followed by definitive TKA [10]. The extensor mechanism is usually short and the vastus medialis is atrophic. Lateral retinacular release with vastus medialis advancement is usually insufficient, but iliotibial band release, quadriceps tendon lengthening, and medial patellofemoral ligament augmentation may be necessary for patellar tracking. In our case, we could not reduce patella tracking because of the greater severity of deformity of bilateral knee.

CDP is associated with shortening and contracture of quadriceps muscles. TKA prosthesis implantation would be associated with an increased distance between the quadriceps and tibial tuberosity, and the quadriceps will become shortened. In a knee with CDP, the quadriceps muscle, the patella, and the patellar tendon pass through the shortest path and the quadriceps muscle does not lengthen during flexion. Therefore, the patella could not be reduced fully and the angle was restricted.

We chose first to reposition the dislocated patella because of the importance of active extension of the knee in walking. The patient could finally extend the left knee fully, but he was not satisfied because of the loss of flexion angle. Therefore, in TKA on the other knee he wished to retain the same level of flexion as before the operation. We performed TKA on the right side without reducing the dislocated patella. He was satisfied with pain relief and flexion in the right knee.

In conclusion, TKA is a useful procedure for osteoarthritis of the knee in association with CDP, but it is difficult to manage this condition. It can be handled with good preoperative planning with regard to whether the patella is or is not reduced, and if necessary how to realign the extensor mechanism.
